# Supplementary material for: Rationale, conceptual issues, and resultant protocol for a mixed methods Person Trade Off (PTO) and qualitative study to estimate and understand the relative value of gains in health for children and young people compared to adults
Source: PLoS One. 2024 Jun 3;19(6):e0302886. doi: 10.1371/journal.pone.0302886 (PMC11146702; doi:10.1371/journal.pone.0302886)
Supplement: S1 Table — (DOCX) [file pone.0302886.s001.docx]

**S1 Table: Maximum sampling quotas for the ORU online sample**

|  | Male | Female | Non-binary | Total |
| --- | --- | --- | --- | --- |
| Age |  |  |  |  |
| Age 16-24 | 160 | 160 |  |  |
| Age 25-34 | 204 | 204 |  |  |
| Age 35-44 | 190 | 190 |  |  |
| Age 45-54 | 190 | 190 |  |  |
| Age 55-64 | 190 | 190 |  |  |
| Age 65-74 | 130 | 130 |  |  |
| Age 75+ | 100 | 100 |  |  |
| Total |  |  | 100 |  |
|  |  |  |  |  |
| Highest qualification |  |  |  |  |
| Degree |  |  |  | 800 |
|  |  |  |  |  |
| State/Territory |  |  |  |  |
| NSW |  |  |  | 1000 |
| Victoria |  |  |  | 1000 |
| Queensland |  |  |  | 600 |
| South Australia |  |  |  | 400 |
| Western Australia |  |  |  | 500 |
| Tasmania |  |  |  | 200 |
| ACT |  |  |  | 200 |
| Northern Territory |  |  |  | 200 |

*Qualification data based on 2022 release of 2021 census. The percentage in Australia with a degree is: Aged 15-74 Female 34.2% Male 28.4%. Australian Bureau of Statistics. Education and Work, Australia [Internet]. Canberra: ABS; 2022 May [cited 2023 Feb 1]. Available from: https://www.abs.gov.au/statistics/people/education/education-and-work-australia/latest-release*

*Age and gender based on ABS Census of Population and Housing: Population data summary, 2021. Australian Bureau of Statistics. Population: Census [Internet]. Canberra: ABS; 2021 [cited 2023 Feb 1]. Available from: https://www.abs.gov.au/statistics/people/population/population-census/2021*

*State/Territory quotas are lenient and designed to ensure some geographical mix rather than geographical representation.*
