# Supplementary material for: Rationale, conceptual issues, and resultant protocol for a mixed methods Person Trade Off (PTO) and qualitative study to estimate and understand the relative value of gains in health for children and young people compared to adults
Source: PLoS One. 2024 Jun 3;19(6):e0302886. doi: 10.1371/journal.pone.0302886 (PMC11146702; doi:10.1371/journal.pone.0302886)
Supplement: S2 Table — (DOCX) [file pone.0302886.s002.docx]

**S2 Table: Issues raised with the survey pilots and how they were addressed**

| Issues raised in the pilot | Steps taken to amend the survey |
| --- | --- |
| The introduction was a bit confusing – would we consider a video? | A video was added explaining the PTO questions and working through an example. |
| The practice question relating to the choice between parks was confusing because this was framed as a personal rather than decision maker choice. | The practice question was removed in favour or a video which showed a practice question based on a comparison between older ages which are not included as part of the actual study. |
| The justification question does not include anything about prioritising adults because they need to work, or prioritising adults because they have dependents, or prioritising children because they have not had a chance to live a long life. | Included as additional options to this question. |
| The three response options for the justification question are confusing. | The response options to the justification question (used/relevant but not used/not relevant) were simplified to – were they used yes/no. |
| It is unclear if parents’ perspective should be considered based on the introductory text | Some of the original text referred to carers and parents which could be confusing if the adults (40 year olds) are also parents. The text was clarified |
| The initial explanation of ‘parents health and wellbeing is considered another way’ is confusing. | The framing of the question was slightly altered to state an assumption that carers health and wellbeing was the same between both programmes. |
| When children are mentioned in the attitude questions it is not clear what age this refers to. | Clarification was added that children refers to 18 and below. |
| There is some lack of clarity regarding the counterfactual for the temporary health option PTO “If I choose an option will that mean that we the other group will be living with the disease or does it mean that they will die now?” | Improved the clarity of explanation around the counterfactual in the text and introductory video. |
| Font size differs within and across some questions. | Font size made consistent. |
| In the questions where it says ‘Prevents a two-year illness which would result in chronic pain’ it was unclear if this mean the illness the treatment prevents is a chronic pain or the after effect of the treatment is chronic pain? | Question altered to state ‘Prevents a 2-year illness which has the symptom **pain** after which they would return to normal health with no long-term consequence’ |
| Unexpected ordering of the iterative routing and incorrect labelling. | Routing of iterative questions corrected and checked and labels amended. |
| Selecting smallest possible group size, which was initially 10. | Added an additional iterative question (1 vs 100) for respondents preferring 10 at the 10 vs 100 choice to help distinguish between very strong preference from lack of willingness to trade. |
| The questions which included life expectancy and age differences were interpreted as if life expectancy differences were caused by the treatment program (hence the largest life expectancy chosen) | These questions were intended to identify whether people had preferences towards prioritising those with shorter life expectancies separately to age but for some people they were not working as expected. These questions were removed. |
| The question on confidence in answers (How confident are you in your answers to these questions? (very confident / somewhat confident / not very confident) was answered negatively by those who spent a lot of time and care with answers and considered it impossible to be confident without additional information e.g. did the patients have dependent children | This question was removed due to difficulties in interpreting people’s motivation for their response. |
| The question asking about serious illness of family members was taking time for respondents to answer and some raised questions such as should grandparents or parents who have died be included (one very old respondent reflected that it would be obvious that her parents must have died so the question must not refer to that) | The question on serious illness of close family members was removed leaving only the question relating to the respondent and their children. |
| When comparing to a 40 year old a number of pilot respondents justified their responses based on the fact that 40 year olds are likely to be caring for young children. | Additional adult age category (age 55) was included which is less likely to be associated with caring for small children, and respondents randomised to either adult age. Additional funds were identified to maintain the sample size for each age comparison. |
