# Supplementary material for: Rationale, conceptual issues, and resultant protocol for a mixed methods Person Trade Off (PTO) and qualitative study to estimate and understand the relative value of gains in health for children and young people compared to adults
Source: PLoS One. 2024 Jun 3;19(6):e0302886. doi: 10.1371/journal.pone.0302886 (PMC11146702; doi:10.1371/journal.pone.0302886)
Supplement: S3 Table — (DOCX) [file pone.0302886.s003.docx]

**S3. Table: Survey attributes**

| Person Trade Off (PTO) detail | Questions | Participant will be asked: |
| --- | --- | --- |
| Young age group (13 categories) | 1 month, 2, 4, 6, 8, 10, 12, 14, 16, 18,20, 22, 24 [ vs age 40 or 55] | One age randomly selected per respondent which will be used in all questions. This randomisation will use the balancing facility within Qualtrics to ensure coverage of all age groups. One randomly selected adult age from either 40 or 55. |
| Health gain context | - Add 2 years of life - Add 5 years of life - Remove a temporary mental health problem (distress low mood and anxiety) - Remove a temporary physical health problem (pain) - Remove a temporary physical health problem (walking or moving about) | 5 questions |
| Additional chaining question | Only the question on life extension (for 2 years) will be used to test ratio level consistency of preferences. This will involve chaining using 2 age comparisons  Given age vs Given age +/- 10 years  Given age +/- 10 years vs 40 | 2 questions |
| Number of patients in each program | Starting position is 100 patients in each group for every question. |  |
| Randomisation of screen position | The youngest age will appear on the left of the screen for 50% of responders and the right for 50% of responders (selected randomly). For ease of comprehension this will be consistent throughout each individual respondent’s questions. |  |
| Randomisation to an arm of the study with/without equivalence option | Participants will be randomized to being offered the option of equivalence or not throughout. The equivalence option (“no preference”) will always be seen in the far-right position. |  |
| Number of iterations | Each PTO question contains the initial choice plus up to 3 subsequent iterative questions less if equivalence is show and is chosen before the final question. An additional iterative question is shown if they prefer 10 over 100 in the final question. |  |
| Demographic questions: | Age, Gender, Education level, Parenthood status, Ethnicity (country of birth and Aboriginal or Torres Strait Islander origin, language mostly spoken at home), Employment status, State/Territory, Experience of serious illness in self or child. |  |
| Forcing choices |  | All questions will be required to be completed to allow progression, and an error message will appear requesting input if responders click on the forward arrow without completing the questions. For demographic questions – a ‘prefer not to say’ option will be included. |
| Introductory video | An introductory video will show the motivation of the study and work through an example PTO question. | Respondents will be required to watch this video and they will not be able to progress forward on the survey until the minutes of the duration of the video has passed. |
