# Supplementary material for: Rationale, conceptual issues, and resultant protocol for a mixed methods Person Trade Off (PTO) and qualitative study to estimate and understand the relative value of gains in health for children and young people compared to adults
Source: PLoS One. 2024 Jun 3;19(6):e0302886. doi: 10.1371/journal.pone.0302886 (PMC11146702; doi:10.1371/journal.pone.0302886)
Supplement: S4 Table — (DOCX) [file pone.0302886.s004.docx]

**S4 Table: Comparison of forced versus unforced choice for an example context and adult age of 40.**

|  | Not offering equivalence | | | | Offering equivalence | | | Combined | | | Test of offering equivalence or not | |
| --- | --- | --- | --- | --- | --- | --- | --- | --- | --- | --- | --- | --- |
|  | n | Ratio of means | Median of ratios & IQR | n | | Ratio of means | Median of ratios & IQR | n | Ratio of means | Median of ratios & IQR | Ratio of means test | Median of ratios test |
| 0 vs 40 |  |  |  |  | |  |  |  |  |  |  |  |
| 2 vs 40 |  |  |  |  | |  |  |  |  |  |  |  |
| 4 vs 40 |  |  |  |  | |  |  |  |  |  |  |  |
| 6 vs 40 |  |  |  |  | |  |  |  |  |  |  |  |
| 8 vs 40 |  |  |  |  | |  |  |  |  |  |  |  |
| 10 vs 40 |  |  |  |  | |  |  |  |  |  |  |  |
| 12 vs 40 |  |  |  |  | |  |  |  |  |  |  |  |
| 14 vs 40 |  |  |  |  | |  |  |  |  |  |  |  |
| 16 vs 40 |  |  |  |  | |  |  |  |  |  |  |  |
| 18 vs 40 |  |  |  |  | |  |  |  |  |  |  |  |
| 20 vs 40 |  |  |  |  | |  |  |  |  |  |  |  |
| 22 vs 40 |  |  |  |  | |  |  |  |  |  |  |  |
| 24 vs 40 |  |  |  |  | |  |  |  |  |  |  |  |
