# Supplementary material for: Rationale, conceptual issues, and resultant protocol for a mixed methods Person Trade Off (PTO) and qualitative study to estimate and understand the relative value of gains in health for children and young people compared to adults
Source: PLoS One. 2024 Jun 3;19(6):e0302886. doi: 10.1371/journal.pone.0302886 (PMC11146702; doi:10.1371/journal.pone.0302886)
Supplement: S1 File — (DOCX) [file pone.0302886.s005.docx]

**S1 File: Analysis steps adopting a thematic approach**

**Step 1** **Transcription:** Verbatim transcription will be undertaken using an automated transcription service. Transcripts will be uploaded to NVivo. The demographic information collected within the main surveys will also be uploaded to NVivo.

**Step 2 Familiarization with the data**: Researchers will check and correct the transcripts whilst listening to the recording to support familiarization. Important nuance of participant speech such as pausing, or hand jesters will be added to the transcript. Researchers will also check any interviewer notes that accompany the recording. Whilst listening to the recordings the researcher will create a memo for any initial responses they may have to the data.

**Step 3 Coding:** The first two transcripts will be coded by at least two researchers (independently) line by line using NVivo (adding ‘nodes’ into NVivo) to highlight broad themes in the interviews, with text linking to one or more codes where appropriate. Coding will pay particular attention to text which relates to the study objectives.

**Step 4 Developing the framework:** Researchers will discuss their coding on the first two transcripts and agree an initial analysis framework (which will include main themes and sub-themes as a coding tree). Steps 3 and 4 will be repeated for two further transcripts, using new codes where appropriate. This initial coding framework will be shared and discussed with the broader team. A further two transcripts will be coded independently by both researchers. They will meet to discuss agreement/variability in their coding and potential additions to the coding and revisions to the analysis framework (such as merging or expanding codes and creating hierarchies). This step will be repeated, with two further transcripts being independently double coded. Where different interviewers have conducted the interviews a mix of their transcripts will be coded up to this point.

Analysis will begin as soon as interviews have been conducted and coding discussion will include discussions on whether saturation has been reached and documenting evidence for this.

**Step 5 Applying the coding framework:** The remaining interviews will be coded by one researcher only. After each additional 8 interviews the researchers will meet for a further discussion and consideration of amendments to the coding and analysis framework. If the analysis framework is amended new versions of transcripts that were analysed at the start of the process will be re-coded.

**Step 6 Thematic analysis:** We will use NVivo capability to support the thematic analysis (e.g. word frequency, cluster analysis (O’Neil, 2012)). We will explore potential links between themes and the demographic information.

**Step 7 Interpretation:** The write up will summarise key components of the content analysis, including both consensus views and outliers. Core themes will be reported, and direct quotations will be taken from the transcripts to provide illustrative data.
