# Supplementary material for: Rationale, conceptual issues, and resultant protocol for a mixed methods Person Trade Off (PTO) and qualitative study to estimate and understand the relative value of gains in health for children and young people compared to adults
Source: PLoS One. 2024 Jun 3;19(6):e0302886. doi: 10.1371/journal.pone.0302886 (PMC11146702; doi:10.1371/journal.pone.0302886)
Supplement: S2 File — (DOCX) [file pone.0302886.s006.docx]

**Topic guide for ‘think aloud’ interviews accompanying the PTO survey**

**1. Welcome**

Interviewer introduces themselves

Interviewer asks questions unrelated to the research to put participant at ease and establish rapport

- *How has your day been?*
- *Where are you calling in from?*
- *How’s the weather there?*
- *Etc.*

**2. Introduction and checking consent**

Participants should already have received and signed consent form prior to the interview allowing participants ample time to consider the information provided and make an informed choice to consent. Interviewer will check that participant has signed consent form prior to interview commencing.

If they have not signed:

The interviewer will interviewer will share screen and allow participants time to read the information (PLS) and will sign on behalf of the participant if they agree.

If they have signed already:

*Before we start, I would like to check that you had a chance to look over the information sheet that we provided you via email. This was the document that explained all about the research project. Did you have any questions about the research project – or anything you would like to ask about?*

Everyone: Interviewer will give a brief recap on the research aims

*Great – so just to recap – this study is all about comparing health improvements for children versus for adults. The questions ask you to make choices between treating different groups of patients – so we can find out what you think about treating people of different ages. There are no right or wrong answers.*

*Just so you know you are free to withdraw at any time – just let me know - and you do not have to offer a reason.*

*Before we start I just want to let you know that I’m about to press record – this is so we can re-listen to the interview afterwards as it is difficult for me to take good notes while doing the interview. Some of the things you say during the interview may be used in a quote for our report, but it won’t be possible to identify you.*

*Do you have any questions? Are you happy to continue?*

**3. Introduction to think aloud**

*We’d really like to understand what you are thinking when you see the question in this survey.*

*So while you answer the questions, I would like you to talk me through your thought process – just saying whatever is coming into your head. There are no right or wrong answers or thoughts here.*

*As well as being interested in your answers to the questions today we are also interested* **how***you are thinking about the tasks – so which bits of the screen stood out most to you, what did it make you think about – that type of thing.*

*We tend to call this ‘thinking aloud’. I’ll give you example of what we mean by ‘thinking aloud’ - imagine I was asked to choose between a cat or a dog – and think aloud while I did so – I might say something like this….*

*“Well it depends - cat’s may have to live inside at least at night which might be tricky, but also dogs can’t go in the National Park, a cat would be cheaper but not as much of a companion – actually I’m not really sure how much a dog would cost and maybe it depends on the size of the dog - and guests might be allergic but actually you never said owning a cat – maybe it was just choosing between which is the better animal, which is clever or which is cuter…”*

*So – you see it doesn’t need to be particularly structured – it’s just saying the things that pop into your head.*

[Note: interviewer to edit above as feels comfortable for them to say]

**4. Survey**

During the survey the interviewer will note down their answers to each of the questions on the prompt sheet. This is so that they can easily refer to the respondent’s previous answers.

*I’m going to share my screen now and we’ll watch an introductory video that explains the survey and talks through an example then we’ll work through the survey questions together.*

Interviewer to run the introductory video.

*Do you have any questions after watching that?*

Interviewer move onto PT) questions and encourage respondent to think aloud

- *What are you thinking now?*
- *What made you choose that option?*
- *Can I just check – when you said XXXXX did you mean XXXX*

Note: need to take care not to lead participants

- *Why did you think that the health gain to that group was more valuable?*

If the respondent asks for clarification prior to giving a response the interview will say:

*Before I say anything too much – can I just ask what you would have assumed or guessed if I wasn’t here – the reason I ask that is we want to know what people might do if they were working through the survey online by themselves.*

Interviewer to take opportunity to extend the survey questions to delve more into respondents’ views

- OK so you think it would be more valuable to treat X year olds. Would you think the same for different ages of children or young people – say if we were thinking about 1 year olds instead (the interviewer will select an age (<24) that is quite different to the initial question age, this will also including babies 1 month old)

Interviewer to flag any differences between the PTO answers and the question context.

- *That’s interesting – you are giving [similar / different] answer in this different context – before we were thinking about [extending life/avoiding pain/improving mobility/avoiding depression]*
- *Would you think the same for other types of health gain – like improvements in being able to look after yourself? Are there other aspects of quality of life where you might wish to treat children or teenagers differently?*
- Are there circumstances where you might think differently (e.g. very poor quality of life, being close to death)?
- (For those who choose equivalence on the first choice) *– If the survey question had only let you have the options A and B which would do you think you would have chosen? - Why?*

Interviewer to flag any differences between the PTO answers and the answers to the attitudinal questions.

- *That’s interesting – earlier it seemed you favoured treating patients who were X years old but on this question you said “x” - do you think being asked questions in this way (referring to the attitudinal questions) gets you to think about different kind of things as when we asked you questions using the different numbers of patients?*

Interviewer to provide positive feedback at the end of the survey

*- Excellent that’s the end of the survey – you did a great job in talking through your views*

**5.0 Semi-structured discussion & debrief**

*This final part of the interview is more of a discussion where you get a chance to tell us what you thought of the survey and how people are likely to answer it.*

*Firstly can I ask a couple of things about the actual survey*

- *Were there any questions you found difficult to answer for any reason?*
- *Did you find the instructions clear in the survey?*
- *Were there any questions that made you feel uncomfortable?*

*Now can we talk a bit about how decision makers might use these answers.*

- *How do you feel about your views being used to support decision making?*
- *Do you think that your views are shared widely across society?*
- *We have spoken to people who think that [child/adults] should be prioritised - do you think that is a reasonable view – or are they missing something important?*

*Is there anything else you would like to tell us about the survey?*

**6. conclusion**

[Another chance to provide positive feedback]

*That brings us to the end of the interview.*

*Do you have any questions about anything that we went through?*

*Thank you very much for your time, the information you have provided is extremely valuable and will help us to understand how people feel about prioritising treatments for children- and inform policy makers.*

*I appreciate that you took time out of your day to speak with me and I’m extremely grateful.*

*Have a nice day,*

*Nice to meet you,*

*Etc.*

INTERVIEWER TO COMPLETE DURING INTERVIEW

Date:

Time:

Interviewer:

|  | Age comparison (left vs right) | Context: (LE2, LE5, Pain, Distress, Mobility) | Final PTO | Reason |
| --- | --- | --- | --- | --- |
| 1. |  |  |  |  |
| 2. |  |  |  |  |
| 3. |  |  |  |  |
| 4. |  |  |  |  |
| 5. |  |  |  |  |
| 6. |  |  |  |  |
| 7. |  |  |  |  |
| 8. |  |  |  |  |

|  | Value questions | Children/Adults/Same | Reason |
| --- | --- | --- | --- |
| 1. | For medical care that improves quality of life temporarily |  |  |
| 2. | For medical care that extends length of life |  |  |

|  | Value questions | Fair/Not sure / Unfair | Reason |
| --- | --- | --- | --- |
| 3. | If the Australian governments were willing to pay more for a treatment for children compared to adults which gave the identical health gain – what would you think? |  |  |
