# Supplementary material for: Rationale, conceptual issues, and resultant protocol for a mixed methods Person Trade Off (PTO) and qualitative study to estimate and understand the relative value of gains in health for children and young people compared to adults
Source: PLoS One. 2024 Jun 3;19(6):e0302886. doi: 10.1371/journal.pone.0302886 (PMC11146702; doi:10.1371/journal.pone.0302886)
Supplement: S3 File — (DOCX) [file pone.0302886.s007.docx]

INTERVIEW PROTOCOL & RISK MANAGEMENT PLAN

Project title: Valuing health care for children, adolescents and young adults compared to valuing health care for adults

Contents

[1 Steps to take prior to the interview 1](#_Toc131176084)

[2 Running the interview 2](#_Toc131176085)

[3 Troubleshooting during interview 2](#_Toc131176086)

[3.1 What if the participant doesn’t enter/arrive at the interview? 3](#_Toc131176087)

[3.2 What if we have internet problems? 3](#_Toc131176088)

[3.3 What if the participant enters the meeting without a camera? 3](#_Toc131176089)

[3.4 What if the participant has a bad microphone/is in a noisy place and I can’t hear them? 3](#_Toc131176090)

[3.5 What if the participant becomes angry when faced with the PTO task? 3](#_Toc131176091)

[3.6 What if the participant becomes upset or stressed with the PTO task or interview questions? 4](#_Toc131176092)

[3.7 What if the participant seems very confused? 6](#_Toc131176093)

[3.8 What if the participant is not paying any attention? 6](#_Toc131176094)

[3.9 What if the interview is taking longer than expected? 6](#_Toc131176095)

[4 Steps to take after the interview 6](#_Toc131176096)

# Steps to take prior to the interview

1. Check your IT set up ensuring that your microphone and camera are working for online interviews and room booking/card access for in person interviews.
2. Using the interview spreadsheet, check that the participants you are interviewing have completed the consent form. If not, you will need to first go to the consent form and talk them through it (see emailed consent form link). If conducted online you will need to complete select the answers as the participant responds. [It is important that participants give genuine informed consent so don’t rush this. They can reschedule if they want more time to reflect on the content of the interview before giving consent]
3. Ensure that you have a copy of the interviewer script ready.

# Running the interview

Have the Qualtrics Interview survey ready. Close other screens on your PC.

Share zoom screen (you will need to click the box on the zoom share screen window (bottom left) to share the sound in order for the sound on the video to play).

Ensure that participants know they can stop the interview at any time if they wish. Be prepared to talk through how the interview has made them feel.

Set the recording at the start of the interview (after consent has been given). This **must be saved onto your computer** and not to the cloud due to data protection issues.

Do not assume that participants can read – they made need help.

For younger participants please be particularly vigilant on whether they are displaying any indication that the interview is making them uncomfortable.

Please work through the qualitative script using the interview prompts or similar phrases.

If it is helpful, during the interview take down notes of their responses so that earlier responses can be referred to during the later part of the interview.

If they appear to be inconsistent in their responses gently probe without making them feel uncomfortable.

Encourage ‘think aloud’ throughout the interview using the suggested prompts on the script.

If they ask for clarification – please first ask what they would have assumed and done if you had not been there.

Note that each of the initial PTO questions has quite a long introduction – this arises in part due to the randomisation of the order of questions. For some you may be able to skip the introduction and just highlight aspects in the PTO tables that have changed.

# Troubleshooting during interview

## 3.1 What if the participant doesn’t enter/arrive at the interview?

Unfortunately, it is quite common for participants to not show up despite having booked an interview.

Contact Lea and CRNRStone research and let them know. Keep a close eye on your email in case anyone is trying to contact you.

## 3.2 What if we have internet problems?

If you find that video is sticking, or you can’t hear the participant because of the internet:

- consider turning your camera off first and if this does not solve the problem, then ask the participant to turn their camera off. You may have to turn the camera off for them.
- Move closer to your router or ask them whether it is possible to move closer to their router if they are using WIFI
- If that does not work, suggest they rearrange the interview time.

## 3.3 What if the participant enters the meeting without a camera?

Ask the participant if their device has a camera and confirm that they are happy to turn it on. Explain that it is important for you to be able to see them when explaining and getting responses.

If they do not have a camera, ask them to consider also dialing into the interview using their phone for a camera and setting it to mute.

## 3.4 What if the participant has a bad microphone/is in a noisy place and I can’t hear them?

Check that sound is set correctly on your end, increase the volume if appropriate. Explain that you can’t hear them and ask if they have access to a headset to use on their device. If they do not, you may need to ask them to position their device closer to them (sometimes this helps) or whether they can move to another device.

If the problem is background noise – ask them whether they can move to another room.

You will need to be able to hear the participant so if you are unable to do so – you may have to end the interview and tell them that Stable Research will reschedule.

## 3.5 What if the participant becomes angry when faced with the PTO task?

Firstly, try and find out more about their views and what aspect of the task they think is inappropriate or offensive.

Participants might find the questions without a ‘no preference’ option most problematic. You can ask them whether they would have picked a no preference option if it had been offered. And tell them that later on in the survey there is an option to comment and that you will note that.

Reassure them along the lines of:

“That’s a really valid view to have - just to say this is set up to be an artificial task – we just use it to try and find out how strongly people hold particular views about prioritizing health care. It’s not meant to reflect real health care choices. That said, when decision makers have a fixed health care budget it is the case that they have to make difficult choices and can’t fund everything. Under a situation where the budget could only fund one program which do you think it should be?

## 3.6 What if the participant becomes upset or stressed with the PTO task or interview questions?

**Protocol for managing participant distress**

**Resources:** Resources to discuss with participant if they are experiencing distress

- [beyondblue](https://www.beyondblue.org.au/) – Telephone 1300 22 4636 www.beyondblue.org.au
- [lifeline](http://lifeline)  Telephone 13 11 14 [www.lifeline.org.au](http://www.lifeline.org.au)
- Kidsline (<25 years) Telephone 1800 551 800 <https://kidshelpline.com.au/>

Depending upon location, discuss access local services – visit GP or call local service help line for state funded support:

- Victoria <http://www3.health.vic.gov.au/mentalhealthservices/> (find appropriate number based on location of participant
- ACT Access Mental Health on [1800 629 354](tel:1800629354) or [02 6205 1065](tel:0262051065)
- NSW 1800 011 511
- NT [1800 682 288](tel:1800682288)
- **Queensland** [1300 MH CALL (1300 642255)](https://www.qld.gov.au/health/mental-health/help-lines/1300-mh-call)
- Tasmania [1800 332 388](tel:1800332388)
- South Australia [13 14 65](tel:131465)
- Western Australia: [1800 437 348](tel:1800437348)

## 3.7 What if the participant seems very confused?

Participants should be able give genuine informed consent throughout the interview if they seem very confused you will need to make a decision on whether it is appropriate to continue. You can either just tactfully draw the interview to a close or suggest it is rescheduled.

## 3.8 What if the participant is not paying any attention?

It will be very poor-quality data if they are not paying attention, and this is particularly a risk with online interviews.

Ask if they would prefer to reschedule if this has turned out to be an inconvenient time for them. If they want to continue you may need to engage in some more conversation to re-engage them. If they are still not engaging you will need to stop the interview.

## 3.9 What if the interview is taking longer than expected?

We need to keep to the 50 minutes.

If participants are going slow this may be a very good thing – their motivations and explanations are more important than their actual answers.

If you think there is a risk of not completing, you may tell the participant that you are going to skip through some of the questions, due to the validation criteria you must answer but these answers but they will be deleted afterwards. Keep a good record of anything you skip through so that data can be removed and follow up on this after the interview.

Please ensure that the participants are not kept after their allocated time and that they do not feel rushed at the end.

# Steps to take after the interview

After the interview, please use the ‘interview spreadsheet’ to state whether the participant attended the interview to enable release of their reward. Please add any comments relating to the interview e.g. technical problems.

The transcript and recording needs to be saved onto the secure MSPGH project folder. Please save using the file name ‘Interview recording interviewer, date and start time’ (using Melbourne time zone)

e.g. Interview recording Tessa 2023-03-27 18-30

e.g. Interview transcript raw Tessa 2023-03-27 18-30

Please ensure any recordings and transcripts are deleted from your zoom folders once they are saved to the project secure drive.

IF YOU ARE IN A POSITION WHERE YOU ARE NOT SURE WHAT TO DO - DO WHAT IS IN THE BEST INTERESTS OF THE PARTICIPANT
