# Supplementary material for: Rationale, conceptual issues, and resultant protocol for a mixed methods Person Trade Off (PTO) and qualitative study to estimate and understand the relative value of gains in health for children and young people compared to adults
Source: PLoS One. 2024 Jun 3;19(6):e0302886. doi: 10.1371/journal.pone.0302886 (PMC11146702; doi:10.1371/journal.pone.0302886)
Supplement: S4 File — (DOCX) [file pone.0302886.s008.docx]

**Topic guide for Focus Groups**

**[DRAFT TO BE FINALISED AFTER INTERVIEWS & SURVEY DATA ANALYSED]**

**0. Checking consent**

Researchers will check consent documentation with all members prior to the start of the focus group.

**1. Welcome & introductions (about 5 mins)**

Researchers welcomes and introduces themselves

Opportunity for all members to provide an introduction.

**2. Introduction and aims of the session (about 5 mins)**

Researchers to present

- Explaining the purpose of the research.
- Setting out the format and guidelines for the session.

**3. Presentation of the study and its findings (about 15 mins)**

Researchers to present

- PTO survey
- Qualitative interview findings
- PTO results

**4. Discussion of the findings & their value for decision making in healthcare (about 60 minutes)**

Now we’d love to hear from you and what you think about this study.

We will try and understand everyone’s different opinions – there are no right or wrong answers.

Led discussion on

a) PTO method & survey:

If we think about the choice questions like this one on the screen [show on screen] – did this seem a reasonable question to ask people? – and a useful way to get at people’s views?

We also asked some opinion type questions - Did these seem the right questions?

Do you think we overlooked anything important?

We tried to ask a representative sample of the Australian public – Do you think we ask the right people or should some people get more say in this type of issue?

b) Initial response to the findings

- Does anything surprise you about the results?

- Do you trust the results of the survey?

c) Who should be deciding the relative weight for children vs adult health

- Some people completed the survey really quickly - Do you think that matters?

- We want to advise decision makers (such as people who decide how to spend medicare funds) on whether the public want to see prioritisation to children - do you think it matters if some people held very different views? Do you think we can just take an average of people’s opinions?

- Do you think children should have more say?

- Should the decision makers decide themselves rather than try and find out the public’s views?

d) What you think a decision maker should learn from the study

- What do you think we can learn from the study?

**6. Conclusion (about 5 minutes)**

[Chance to provide positive feedback]

That brings us to the end of the focus group.

Do you have any questions about anything that we went through?

Thank you very much for your time, the information you have provided is extremely valuable and will help us to understand how people feel about prioritising treatments for children- and inform policy makers.

I appreciate that you took time out of your day to speak with me and I’m extremely grateful.

Have a nice day,

Nice to meet you,

Etc.
