# Supplementary material for: Rationale, conceptual issues, and resultant protocol for a mixed methods Person Trade Off (PTO) and qualitative study to estimate and understand the relative value of gains in health for children and young people compared to adults
Source: PLoS One. 2024 Jun 3;19(6):e0302886. doi: 10.1371/journal.pone.0302886 (PMC11146702; doi:10.1371/journal.pone.0302886)
Supplement: S5 File — (DOCX) [file pone.0302886.s009.docx]

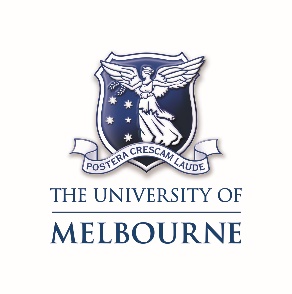

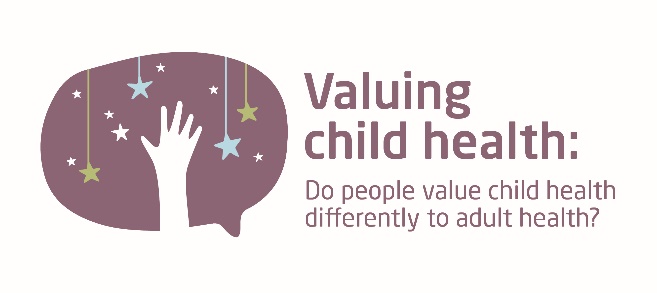


## ***Project:*** ***Valuing health care for children, adolescents and young adults compared to valuing health care for adults***

Dr Tessa Peasgood (Responsible Researcher), School of Population and Global Health
Email: Tessa.Peasgood@unimelb.edu.au

### Introduction

Thank you for your interest in this research project. The following few pages will tell you about the project, so that you can decide if you would like to take part. You may ask questions about anything you don’t understand or want to know more about by emailing Tessa at [Tessa.Peasgood@unimelb.edu.au](mailto:Tessa.Peasgood@unimelb.edu.au)

### What is this research about?

We want to find out whether people feel differently about health treatments which improve the health of children compared to adults. Our findings will be shared with decision makers in government to better inform them about what you (the public) think is the best way to use health care funds.

### Who is running the research project?

The project is based in the University of Melbourne. The project has a team of researchers including health economists, clinicians and health care researchers. This includes Tessa Peasgood, Cate Bailey, Ashwini De Silva and Nancy Devlin from the University of Melbourne, Richard Norman from Curtin University and Rosalie Viney from the University of Technology Sydney.

### What will I be asked to do?

This study will be conducted online and use Zoom, a videoconferencing software that is free for individuals to use (we’ll tell you how to get started with that later). You will need a computer, laptop or tablet with a camera and internet connection to take part.

You will be invited to join a one-to-one interview. During the interview, the interviewer will share their screen showing a set of survey questions. **The interview will take about 50 minutes.** Firstly, you will watch a short video which explains the study and what will be involved.

You will then complete a set of questions where you will be asked to think about the way you value treatments for children compared to treatments for adults. Whilst answering these questions a researcher will ask you to talk about what you are thinking and explore the reasons behind your decisions. There are no right or wrong answers to the survey questions – we just want to know what you think.

We will also ask you some background questions about you and your health – this is just so that we know who we have spoken to and how similar the people we survey are to the Australia population.

Prior to the interview we will ask you to confirm that you are happy to take part by filling in a consent form. You can also tell us on this form whether you would like to be sent a summary of the findings when the research is finished.

### What are the possible benefits?

This research will help health care decision makers in Australia understand how members of the public feel about giving priority to treatments for children compared to adults.

You will be compensated with a $60 as a thank you for your time. An electronic voucher will be sent by CRNRStone Research once we let them know that the interview has been completed.

### What are the possible risks?

Although we expect there are no risks, thinking about children leading short lives or living in poor health may cause distress to some people. You will be able to stop the interview at any time if you wish. The interviewer will be happy to talk through how the interview has made you feel.

We have put a couple of resources at the end of this which might be useful for you if you feel in need of talking to someone after the interview. If you would like to talk to the researcher afterwards to discuss the survey, please email Tessa [Tessa.Peasgood@unimelb.edu.au] or Nancy [Nancy.Devlin@unmelb.edu.au] and we’ll arrange a time.

### Do I have to take part?

Participation is completely voluntary. You can withdraw at any time without giving a reason why. If you do decide to stop the interview any data that has already been collected as part of the interview will be deleted from the study.

### What will happen to information about me?

All information that is collected about you during the research will be kept confidential. The information you give will not be used in any way that could identify you. Your name will not be linked to the data you give us during interview or survey.

We will collect your name on the consent form and your email if you requested a summary of the research findings. This personal information will only be used as a record of your consent and so that we can send out the research findings. It will be stored securely at the University of Melbourne and deleted after 5 years.

The anonymised data may also be used by other researchers. This will be restricted to future projects that are *related* to this project, *or in the same general area* of research as this project. The anonymous data from this research will be archived securely with the University of Melbourne.

The results of the study may be published in academic journals and presented at conferences. Nobody will be able to identify you in any publications.

### Who is funding this project?

This study is funded by the Medical Research Futures Fund (MRFF) through the Australian Government.

### Where can I get further information?

If you would like more information about the project, please contact the researcher; Tessa Peasgood [Tessa.Peasgood@unimelb.edu.au]

### Who can I contact if I have any concerns about the project?

This project has human research ethics approval from The University of Melbourne [2023-24869-37630-4]. If you have any concerns or complaints about the conduct of this research project, which you do not wish to discuss with the research team, you should contact the Research Integrity Administrator, Office of Research Ethics and Integrity, University of Melbourne, VIC 3010. Tel: +61 3 8344 1376 or Email: research-integrity@unimelb.edu.au. All complaints will be treated confidentially. In any correspondence please provide the name of the research team and/or the name or ethics ID number of the research project.

### If the interview has upset you in any way these resources might be useful

[**beyondblue**](https://www.beyondblue.org.au/) – and organisation that aims to increase awareness of [depression](https://www.healthdirect.gov.au/depression) and [anxiety](https://www.healthdirect.gov.au/anxiety) and [reduce stigma](https://www.healthdirect.gov.au/reducing-mental-illness-stigma).

Call 1300 22 4636, 24 hours / 7 days a week Website: www.beyondblue.org.au

[**lifeline**](https://www.lifeline.org.au/) provides 24-hour crisis counselling, support groups and suicide prevention services. Call 13 11 14. Website: www.lifeline.org.au
