# Supplementary material for: Rationale, conceptual issues, and resultant protocol for a mixed methods Person Trade Off (PTO) and qualitative study to estimate and understand the relative value of gains in health for children and young people compared to adults
Source: PLoS One. 2024 Jun 3;19(6):e0302886. doi: 10.1371/journal.pone.0302886 (PMC11146702; doi:10.1371/journal.pone.0302886)
Supplement: Attachment — Submitted filename: Response to reviewers 2024-02-18.docx [file pone.0302886.s011.docx]

We would like to sincerely thank the reviewers for taking the time to re-assess our manuscript and evaluate the changes made. Below we address the remaining concerns (our responses are in red).

Reviewer #1: This is a significant proposal, and the paper is now much clearer and more accessible. Thank you for making these changes. However, the paper is still much fuller than a traditional study protocol, and the extent of your analysis is hidden by the presentation and in particular the title. Conversely, though the title emphasises the PTO approach, this is only introduced quite surreptitiously in section 2.3.

The paper would be more appropriately titled: "Rationale, conceptual issues, and resultant Person Trade Off (PTO) protocol for a mixed methods and qualitative study to estimate and understand the relative value of gains in health for children and young people compared to adults".

Response: We agree that your suggested title more accurately describes the content of the paper and is superior to the current one. We have therefore amended the title in line with your suggestion. Thank you.

In this vein, the key words should be reconsidered and re-ordered.

Thank you for noting this. The current choice of key words are: “Person Trade Off (PTO); valuation health; children; Mixed Methods; social value; Australia; qualitative; online survey; public preferences” We have now amended the order to give less prominence to PTO and included a couple of more general terms to communicate the discussion components included in this protocol.

The key words are now (with additional terms underlined):

“Social value; priority setting; public preferences; Person Trade Off (PTO); valuation health; children; mixed methods; qualitative; online survey; conceptual issues; Australia”
